# Cultivation and Complete Genome Sequencing of *Gloeobacter kilaueensis* sp. nov., from a Lava Cave in Kīlauea Caldera, Hawai'i

**DOI:** 10.1371/journal.pone.0076376

**Published:** 2013-10-23

**Authors:** Jimmy H. W. Saw, Michael Schatz, Mark V. Brown, Dennis D. Kunkel, Jamie S. Foster, Harry Shick, Stephanie Christensen, Shaobin Hou, Xuehua Wan, Stuart P. Donachie

**Affiliations:** 1 Department of Microbiology, University of Hawai'i at Mānoa, Honolulu, Hawai'i, United States of America; 2 Cold Spring Harbor Laboratory, Cold Spring Harbor, New York, United States of America; 3 NASA Astrobiology Institute, University of Hawai'i, Honolulu, Hawai'i, United States of America; 4 School of Biotechnology and Biomolecular Sciences, The University of New South Wales, Sydney, New South Wales, Australia; 5 Dennis Kunkel Microscopy, Inc., Kailua, Hawai'i, United States of America; 6 Department of Microbiology and Cell Science, University of Florida Space Life Science Laboratory, Kennedy Space Center, Kennedy, Florida, United States of America; 7 Kea'au, Hawai'i, United States of America; 8 Department of Oceanography, School of Ocean and Earth Science and Technology, University of Hawai'i at Mānoa, Honolulu, Hawai'I, United States of America; 9 Advanced Studies of Genomics, Proteomics and Bioinformatics, University of Hawai'i at Mānoa, Honolulu, Hawai'i, United States of America; The Centre for Research and Technology, Hellas, Greece

## Abstract

The ancestor of *Gloeobacter violaceus* PCC 7421^T^ is believed to have diverged from that of all known cyanobacteria before the evolution of thylakoid membranes and plant plastids. The long and largely independent evolutionary history of *G. violaceus* presents an organism retaining ancestral features of early oxygenic photoautotrophs, and in whom cyanobacteria evolution can be investigated. No other *Gloeobacter* species has been described since the genus was established in 1974 (Rippka *et al.*, Arch Microbiol 100:435). *Gloeobacter* affiliated ribosomal gene sequences have been reported in environmental DNA libraries, but only the type strain's genome has been sequenced. However, we report here the cultivation of a new *Gloeobacter* species, *G. kilaueensis* JS1^T^, from an epilithic biofilm in a lava cave in Kīlauea Caldera, Hawai'i. The strain's genome was sequenced from an enriched culture resembling a low-complexity metagenomic sample, using 9 kb paired-end 454 pyrosequences and 400 bp paired-end Illumina reads. The JS1^T^ and *G. violaceus* PCC 7421^T^ genomes have little gene synteny despite sharing 2842 orthologous genes; comparing the genomes shows they do not belong to the same species. Our results support establishing a new species to accommodate JS1^T^, for which we propose the name *Gloeobacter kilaueensis* sp. nov. Strain JS1^T^ has been deposited in the American Type Culture Collection (BAA-2537), the Scottish Marine Institute's Culture Collection of Algae and Protozoa (CCAP 1431/1), and the Belgian Coordinated Collections of Microorganisms (ULC0316). The *G. kilaueensis* holotype has been deposited in the Algal Collection of the US National Herbarium (US# 217948). The JS1^T^ genome sequence has been deposited in GenBank under accession number CP003587. The G+C content of the genome is 60.54 mol%. The complete genome sequence of *G. kilaueensis* JS1^T^ may further understanding of cyanobacteria evolution, and the shift from anoxygenic to oxygenic photosynthesis.

## Introduction

*Cyanobacteria* are among the most diverse and successful microbes on Earth. As pioneers of oxygenic photosynthesis they permanently changed Earth's atmosphere by emitting gaseous diatomic oxygen, paving the way for the evolution of aerobic metabolism. *Gloeobacter violaceus* refers to the modern representative of an early branching cyanobacterium that diverged from other cyanobacteria before the emergence of plant plastids [1], [2], and which is thought to be one of the earliest cyanobacteria capable of oxygenic photosynthesis. It is thus considered an intermediary organism due to its primordial characteristics [3]. *G. violaceus* is also unique because it lacks the thylakoid membranes essential in all other cyanobacteria and plant plastids as the sites of light-dependent reactions in photosynthesis [4]. The absence of these membranes from *G. violaceus* has led to speculation that the genus may host the earliest ancestors, or a missing link, in the cyanobacteria lineage [3]. However, no other *Gloeobacter* species has been named since the original description in 1974 [4]. Although the name *Gloeobacter* was not included in the Approved Lists of Bacterial Names in 1980, Rule 28a of the Bacteriological Code would permit revival of the name *Gloeobacter* through proposal of a new species [5]. In this respect, cyanobacteria have historically been described under the *International Code of Botanical Nomenclature* (ICBN) because they are considered ‘cyanophytes’, or plants, but the original description of *G. violaceus* PCC 7421^T^ was not valid under the ICBN because the Latin diagnosis required between 1958 and 2012 was not part of the original *Gloeobacter* publication. A recent change to the ICBN, now termed the *International Code of Nomenclature for algae, fungi, and plants*, removed the requirement for a Latin diagnosis [6]; the genus *Gloeobacter* may still have no standing under this or the original ICBN because the type designated was a living culture, not an herbarium mount. Only three strains described as *Gloeobacter violaceus* have been cultivated, PCC 7421^T^ [4], PCC 8105 [2] and VP3-01 [7]. *G. violaceus* PCC 7421^T^ was isolated from the surface of a limestone rock in Canton Obwalden, Switzerland in 1974 [4], and its genome was sequenced in 2003 [8]. Regardless, the genus *Gloeobacter* has essentially had no standing in bacterial nomenclature for almost forty years, but a recent paper addresses some of the issues described above [9]. The taxonomic status of the *Gloeobacter* is now an especially pressing issue because we report here the isolation of a second species in the genus, *G. kilaueensis* JS1^T^ sp. nov., and the sequencing of its complete genome. We also compare the genomes of JS1^T^ and *G. violaceus* PCC 7421^T^, and discuss the position of JS1^T^ in the *Cyanobacteria*.

While investigating microbial diversity in Hawaiian lava caves we collected samples for molecular and cultivation work from a purple-pigmented epilithic biofilm on the entrance wall of a cave in volcanically active Kīlauea Caldera. Pyrotag and metagenomic libraries revealed phylogenetically diverse taxa, including those with no known cultivated representatives, or which shared low 16S rDNA nucleotide identity with known taxa in the *Archaea* or *Bacteria*, or which affiliated with named taxa, *e.g.*, *Gloeobacter* (unpublished data).

We targeted the putative *Gloeobacter* for cultivation in the laboratory. An axenic culture was not obtained, but extracting and sequencing genomic DNA from a mixed culture provided sequences resembling a low-complexity metagenome, with most sequences derived from the dominant organism (*Gloeobacter*) and few sequences from other organisms. A *de novo* assembly of the sequences provided the complete genome of JS1^T^. Comparing this genome with that of *Gloeobacter violaceus* PCC 7421^T^ provided evidence we had cultivated a new *Gloeobacter*, and insights into the divergence and evolution of *Gloeobacter* from other cyanobacteria. Gene and sequence conservation, synteny, and genome-to-genome distances calculated for both organisms showed JS1^T^ does not belong to *G. violaceus*. Strain JS1^T^ thus represents a novel species in the *Gloeobacter*, for which we propose the name *Gloeobacter kilaueensis* sp. nov. The type strain of *G. kilaueensis* sp. nov. is JS1^T^.

## Results and Discussion

### Sampling, Isolation, and Sequencing

A cloned 16S rDNA sequence (EF032784) sharing 98.65*%* nucleotide identity with that of *G. violaceus* PCC 7421^T^ was detected in a 16S rDNA clone library constructed from community genomic DNA extracted from a purple-pigmented epilithic biofilm in a lava cave in Kīlauea Caldera, Hawai'i (Brown et al., unpubl.). The cave is ∼800 m from the volcanically active Halema'uma'u pit crater. As volcanic activity here increased through 2009, temperatures 25 m into the cave ranged from 35 to 40°C, and relative humidity exceeded 100*%*; condensation formed on all surfaces, and the cave floor was hot to the touch. Immediately below the cave's ∼1 m^2^ ground-level entrance, the air temperature was ∼30°C, and condensation flowed over and dripped from the epilithic biofilm.

Single punch cores (5 mm diameter) of the ∼5 mm thick biofilm were collected aseptically into separate sterile 2 ml cryotubes. In the laboratory, cores were aseptically transferred to a modified BG-11 liquid medium and incubated with shaking at 28°C under 500±20 lx (∼46 fc; ∼9 µE m^−2^ sec^−1^) in a 24 hr light regime; additional cores were streaked or serially diluted and spread on modified BG-11 plates and incubated under the same conditions. All inoculated tubes and plates were wrapped loosely in white tissue paper to attenuate the light reaching the cells. After one month, purple aggregates and purple colonies arose in liquid and on solid media, respectively. At least two heterotrophic bacteria species grew in close association with the purple colonies. The purple colonies appear to develop weakly at 37°C, the highest temperature at which growth was tested.

**Figure 1:**
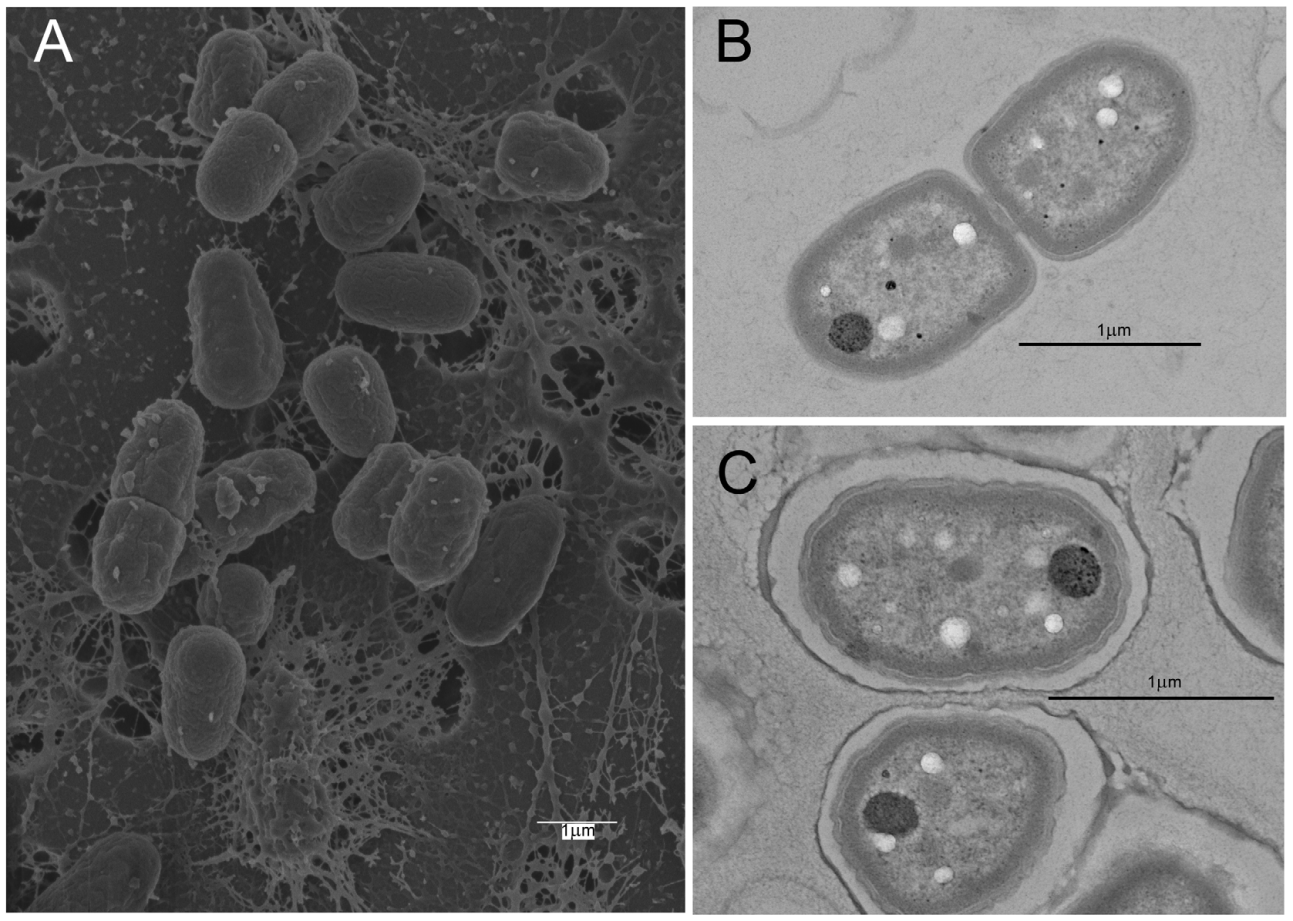
Scanning electron micrograph of *Gloeobacter kilaueensis* JS1^T^ cells grown in modified BG-11 liquid medium. Dividing cells are near the top and left of the image. Note mucilaginous material among the cells. Scale bar is 1 µm.

Prior to constructing paired-end 454 and Illumina libraries, live and Gram-stained cells from cultures in modified BG-11 were observed by epifluorescence and light microscopy to semi-quantitatively gauge diversity through the range of bacteria cell shapes and sizes present. Cells were predominantly uniform, unicellular and autofluorescent, and matched the dimensions (∼3.5×1.5 µm) of *Gloeobacter*; few other cell types were observed (Fig. S1). Scanning electron microscopy (SEM) showed ovoid cells often in mucilaginous sheaths (Fig. 1A), while transmission electron microscopy (TEM) did not reveal thylakoid membranes in the presumed *Gloeobacter* cells (Figs. 1B, C). Major pigment peaks were determined by HPLC in non-axenic cultures of JS1^T^. Of 42 clones in a 16S rRNA gene clone library constructed from genomic DNA extracted from such a culture in BG-11, 40 affiliated with *Gloeobacter*, and one each with *Bradyrhizobium* sp. and *Burkholderia* sp. Cultures of JS1^T^ were deposited in the American Type Culture Collection (BAA-2537), the Scottish Marine Institute's Culture Collection of Algae and Protozoa (CCAP 1431/1), and the Belgian Coordinated Collections of Microorganisms (ULC0316). The holotype of *G. kilaueensis* has been deposited in the Algal Collection of the US National Herbarium (US# 217948).

### Genome assembly and verification

Nucleotide sequences were generated from a single pool of genomic DNA extracted from a non-axenic culture determined by microscopy and a 16S rRNA gene clone library to contain few non-*Gloeobacter* cells. A total of 376,649 pyrosequences (310,136 paired-end and 66,513 singleton reads) and 4,792,504 Illumina reads were generated. The average pyrosequence read length was 199.1 bp, after reads were split into left- and right-paired segments. The average singleton read length was 281.6 bp. Illumina sequences were generated from 400 bp paired-end fragments and comprised 2,396,252 paired-end reads (total, 4,792,504 reads). Sequence data and assembly statistics are listed in Table 1.

**Table 1:** *G. kilaueensis* JS1^T^ genome assembly statistics.

Total number of 454 pyrosequences	376,649
Total number of Illumina sequences	4,792,504
Contigs generated by Newbler	145
Scaffolds generated by Newbler	1
Contigs generated by Celera	83
Scaffolds generated by Celera	66
Contigs generated by Velvet	3,157
Total sequence coverage	93×

**Figure 2:**
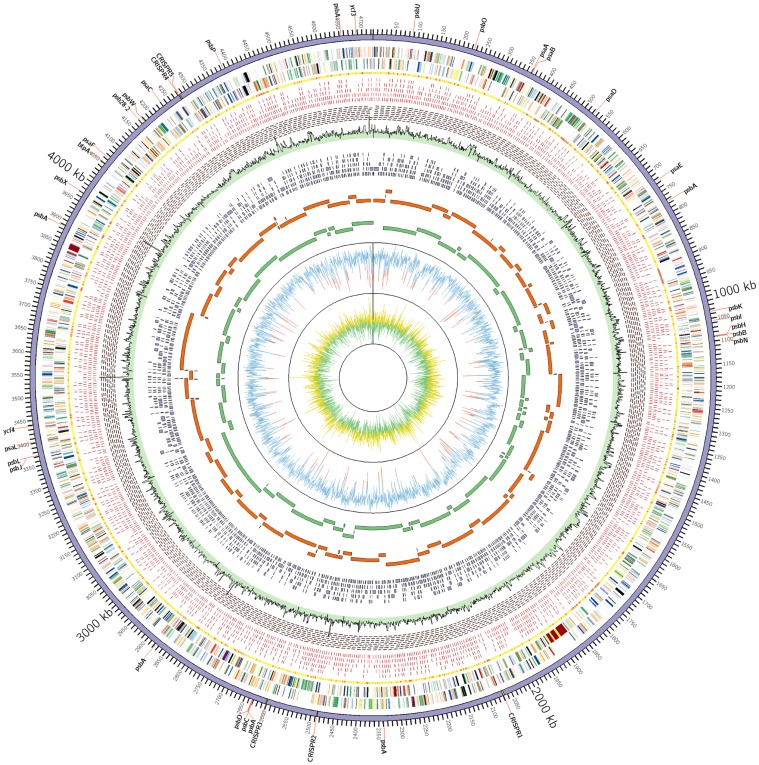
Circular representation of the *Gloeobacter kilaueensis* JS1^T^ genome. From inside out: GC skew (Yellow>0, Green<0), GC percent (Blue>50%, Red<50%), Newbler scaffold contigs, Celera contigs, Velvet contigs (Illumina reads only), read coverage (Combined 454 and Illumina reads sampled for 1,000 bp window. Highest coverage is 368×), minimal tiling clone pairs (shown in red), recruited reads from metagenome, taxonomic rank of top BLAST hit (yellow = *Cyanobacteria*, Red = others, Grey = no BLAST hit), coding regions in minus and plus strands (colored by COG functional categories). CRISPR repeat regions are highlighted in yellow in the outermost circle. Locations of genes involved in photosystems are labeled in the outermost circle.

To prevent contaminant sequences from co-assembling with *Gloeobacter* sequences, 9 kb paired-end 454 sequences were assembled with the Newbler assembler to utilize paired-end constraints. Pyrosequence assembly resulted in a single scaffold of 146 contigs (Fig. 2; Table 1). Illumina sequences were also paired-end sequences with insert sizes of ∼400 bp. All assembled contigs had 9 kb mate pairs linking the contigs (Fig. 2). Each contig produced by Newbler also had paired-end 454 reads spanning the whole contig (Fig. S2). A hybrid assembly using Celera assembler with both 454 and Illumina reads produced 66 contig scaffolds with 83 contigs. The total number of bases in the scaffolds was 4,799,862 bp. Contigs from the Celera assembly were aligned against the Newbler contigs to check discrepancies between assemblies (Fig. 2). Generally, contigs produced by both assembly methods were comparable and complementary, although contig breaks occurred at different positions along the genome.

Newbler contig scaffolds provided a framework to orient the contigs and to close the remaining gaps between the contigs. To aid gap closure, Illumina sequences were independently assembled using the Velvet assembler [10]. The assembly produced 3,157 contigs averaging 1,741 bp (Fig. 2; Table 1). The largest contig was 56 kbp. Velvet contigs were shredded into 500 bp fragments with 250 bp overlapping regions and manually assembled with Newbler contigs by MINIMUS and SeqManII (DNASTAR Inc, Madison, WI) to close the gaps. Amplification by PCR of the remaining gap regions followed by capillary sequencing closed all gaps. To improve the sequence quality of the final assembled contig, Illumina reads were recruited using MUMMER [11]; recruited reads were assembled with the final contig using MINIMUS. This step fixed ambiguous bases introduced by homopolymers present in 454 pyrosequences, and improved the overall quality of the assembled genome sequence.

To verify the assembly and identify potentially misassembled regions in the genome, Newbler and Celera contigs and pyrosequences were aligned against the finished genome with MUMMER. Mate pairs and singletons produced by 454 sequencing were binned using the PhymmBL program [12], [13] to assign taxonomic ranks to these reads. The intention was to bin *Gloeobacter* reads from non-\*Gloeobacter* reads to visualize sequence coverage along the genome. Where sequence coverage for *Gloeobacter*-specific reads dropped, those regions were manually checked and verified by long-range PCRs to confirm the presence of such regions. Five such regions were considered questionable in this respect due to the low coverage of *Gloeobacter* sequences, and because the G+C% dropped below 60% (G+C mol% of the complete genome is 60.54). However, it is important to note that the PhymmBL tool has an accuracy of 78.4*%* in assigning taxonomic rank at genus level, and the algorithm involves comparison with known sequences from GenBank [13]. Given there is just one *Gloeobacter* genome in GenBank, binning could have produced false positives or negatives due to low representation of *Gloeobacter*-specific sequences in the database.

Through BLAST searches, ‘suspicious’ regions appeared largely to contain genes from other organisms (Table S1; Fig. S2). To determine if these regions were in fact part of the JS1^T^ genome, primers were designed to amplify approximately 15 kb fragments that would include them. Nine such long-range PCR reactions and sequencing confirmed these regions are part of the genome and not sequence assembly artifacts (Fig. S3). The complete genome sequence of JS1^T^ has been deposited in GenBank under accession number CP003587.

### Characteristics and features of the *G. kilaueensis* JS1^T^ genome

The JS1^T^ genome comprises 4,724,791 bp, and has a G+C mol% content of 60.54 (Table 2). The GC content of *G. kilaueensis* JS1^T^ (hereafter referred to as JS1^T^) is 1.5*%* lower than that of *G. violaceus* PCC 7421^T^ (hereafter referred to as GVIO). G+C content variations in the chromosome are evident in several regions, including the suspicious regions checked by long PCR, and appear to contain phage-related genes or mobile genetic elements such as transposons.

**Table 2:** General features of the *G. kilaueensis* JS1^T^ genome and comparison with that of *Gloeobacter violaceus* PCC 7421^T^.

	G. kilaueensis JS1^T^	G. violaceus PCC 7421^T^
Size (bp)	4,724,791	4,659,019
G+C mol%	60.5	62.0
Total number of ORFs	4,508	4,430
Protein coding (%)	90.4	89.4
Proteins with known functions	2,245	1,788
Hypothetical proteins	1,642	2,642
Total number of rRNA operons	1	1
Total number of tRNA genes	49	45
Other RNA	8	4
CRISPR repeat regions	5	0

A total of 49 tRNAs, 1 rRNA operon, and 4,508 protein-coding genes were identified in the JS1^T^ genome. The functions of 2862 (63.5%) of the protein-coding genes were predicted; 1655 (36.7%) were annotated as hypothetical proteins, and 313 (6.9%) had no BLAST hits in the Refseq database at an E-value cutoff of 1×10^−5^. About 34% of the proteome has no hits to COGs (Cluster of Orthologous Groups). Protein-encoding genes by COG functional categories were compared to those in the GVIO genome. The top three COG functional categories are cell wall/membrane/envelope biogenesis (5.9%), transcription (4.7%), and amino acid transport and metabolism (4.4%).

### Phage-related genes

Phage are important agents in genetic exchange between different bacteria species, and phage-related regions constitute genomic hotspots in cyanobacteria such as *Prochlorococcus* [14]. Such hotspots or genomic islands contribute as much as 10–30% to the diversity between different bacteria strains [15]. Regions of the JS1^T^ genome mostly in the questionable regions mentioned earlier seem to have been acquired from other organisms. Genes in these regions have no BLAST hits, are from other bacteria, or are of viral origin; of 196 such ORFs identified, 75 have no BLAST hits, and 136 have no known function and are annotated as hypothetical proteins. Among genes of viral origin are some that appear to belong in the Caudovirales, dsDNA viruses with no RNA stage, *i.e.*, phage.

### CRISPR regions

Using the CRISPR Finder tool, 5 CRISPR repeats were detected in JS1^T^ (Table S2). There are no CRISPR repeat regions in the GVIO genome. In addition to CRISPR repeats, CRISPR-associated proteins (Cas1, Cas2, Cas4, and Csc2) were found in the JS1^T^ genome. Cas1 (GKIL_1965), Cas2 (GKIL_1966), Cas4 (GKIL_1964), and Csc2 (GKIL_1961) were found close to CRISPR repeat region 1 (2066878–2070197). Additional copies of CRISPR-associated proteins Cas1 (GKIL_4060) and Cas2 (GKIL_4059) were found close to CRISPR region 5 (4273038–4274931). CRISPR-associated protein, APE2256 family (GKIL_2360), was found close to CRISPR region 2 (2486198–2486962). CRISPR repeats are a type of bacterial immune system that helps them defend against viruses [16], [17]. The presence of phage genes and CRISPR regions in the JS1^T^ genome suggests the epilithic biofilm hosts viruses and bacteriophage that may pose threats to the bacteria. This is an interesting observation because CRISPR regions have also been reported in microbes in hot-spring photoautotrophic mats in volcanically active Yellowstone National Park and this may indicate higher virus activities in geothermally active areas [18].

### Absence of thylakoid membranes

*G. violaceus* PCC 7421^T^ is known to lack thylakoid membranes, and it is this lack of thylakoid membranes that led to intense study of the species on the grounds it may be the missing link in anoxygenic to oxygenic photosynthesis [3], [19]. The absence of thylakoid membranes from *G. kilaueensis* JS1^T^ was confirmed by TEM (Figs. 1B, C), while some of the genes involved in thylakoid membrane formation were not detected in the annotated genome. Genes *sqdB* (encoding sulfolipid biosynthesis protein) and *sqdX* (encoding UDP-sulfoquinovose:DAG sulfoquinovosyltransferase) are required for synthesis of sulfoquinovosyl diacylglycerol (SQDG) which is in turn required for photosystem stabilization (the product SQDB is usually found in thylakoid membranes) in other cyanobacteria, but is absent from *G. violaceus* PCC 7421^T^ [20], [21]. Neither *sqdB* nor *sqdX* were detected in the JS1^T^ genome, as BLASTp searches using *Synechococcus* SqdB and SqdX yielded only weak hits with less than 30% sequence identities at the amino acid sequence level. The Vipp1 protein is also known to be essential for the formation of thylakoid membrane in *Synechocystis* [22] and *Arabidopsis thaliana* [23], and has been detected in *G. violaceus* PCC 7421^T^, but the ortholog in PCC 7421^T^ (which is annotated as phage shock protein, PspA) seems to be missing the conserved C-terminal region in its amino acid sequence, and is not expected to function the same way as *Synechocystis* or plant Vipp1 protein [8]. A copy of the Vipp1 homolog was also detected in the JS1^T^ genome (GKIL 4366 - phage shock protein A, PspA) but is nearly identical to PCC 7421^T^ PspA protein, and also lacks the conserved C-terminal [8].

### Secondary metabolite biosynthesis pathways

*G. violaceus* produces a range of pigments, including β-carotene, oscillol diglycoside, and echinenone [24], [25], and its purple color is thought to result from a low chlorophyll content [4]. HPLC analysis revealed JS1^T^ contains chlorophyll *a* and β-carotene. Although the analysis does not provide a complete profile of the pigments in JS1^T^, the methods are widely used to determine chlorophyll and carotenoid pigments in bacteria. Pathway Tools was used to check secondary metabolite pathways and revealed one for neurosporene biosynthesis, a subclass of *trans*-lycopene biosynthesis I in *Bacteria*.

The *trans*-lycopene biosynthesis I pathway synthesizes *all-trans*-lycopene, a bright red carotenoid pigment usually found in photosynthetic organisms and a precursor of other pigments. *G. violaceus* is known to use bacterial-type phytoene desaturase from this pathway to synthesize major pigments such as ß-carotene and (2S,2′S)-oscillol 2,2′-di(α-L-fucoside), and a minor pigment known as echinenone [24]. Genes for phytoene synthase (*crtB*) and phytoene desaturatase (*crtN*) were found in the JS1^T^ genome. The biosynthesis pathway for neurosporene, a sub-class of that for *trans*-lycopene biosynthesis I, converts *all-trans*-phytoene to *all-trans*-neurosporene; this is utilized by purple non-sulfur bacteria such as *Rhodobacter capsulatus* and *Rh. sphaeroides* to produce the pigment spheroidene, required in the photoreaction center of these bacteria [26]. *Rhodobacter* spp. are of interest because they are capable of anoxygenic photosynthesis, and can function in both aerobic and anaerobic conditions [27].

### Vancomycin resistance genes

The Pathway Tools program predicted that JS1^T^ has a vancomycin resistance pathway, revealed by the presence of *vanB* (GKIL_3597), *vanX* (GKIL_1509 and GKIL_1879), and *serA* (GKIL_0932). GVIO, however, only has a copy of *vanX* (gll1805) and *serA* (gvip294), and is missing *vanB*. Five essential gene products are in fact required for high-level vancomycin resistance, specifically VanR, VanS, VanH, VanX, and either VanA, VanB or VanD [28]–[30]. The vancomycin resistance pathway in JS1^T^ only comprises genes for VanX and VanB, so the strain may not be fully resistant to vancomycin. We investigated the effect of vancomycin on JS1^T^ with BD Sensi-Disc™ Susceptibility Test Discs, containing 30 mg vancomycin per disc, on BG-11. JS1^T^ was not resistant to the vancomycin concentration presented.

### *In silico* DNA-DNA hybridization and determination of species rank

Species definition and delineation in prokaryotes has been discussed at length, and DNA-DNA hybridization (DDH) values ranging from 60–70*%* have historically been the threshold for distinguishing species [31]. However, the availability of complete genome sequences means that *in silico* genome comparisons can replace *in vitro* DDH experiments by Average Nucleotide Identities (ANI), or Genome-to-Genome distances [32]–[34]. For example, this approach delineates closely related *Bacillus* species and strains, and correlates well with *in vitro* DDH [35].

**Figure 3:**
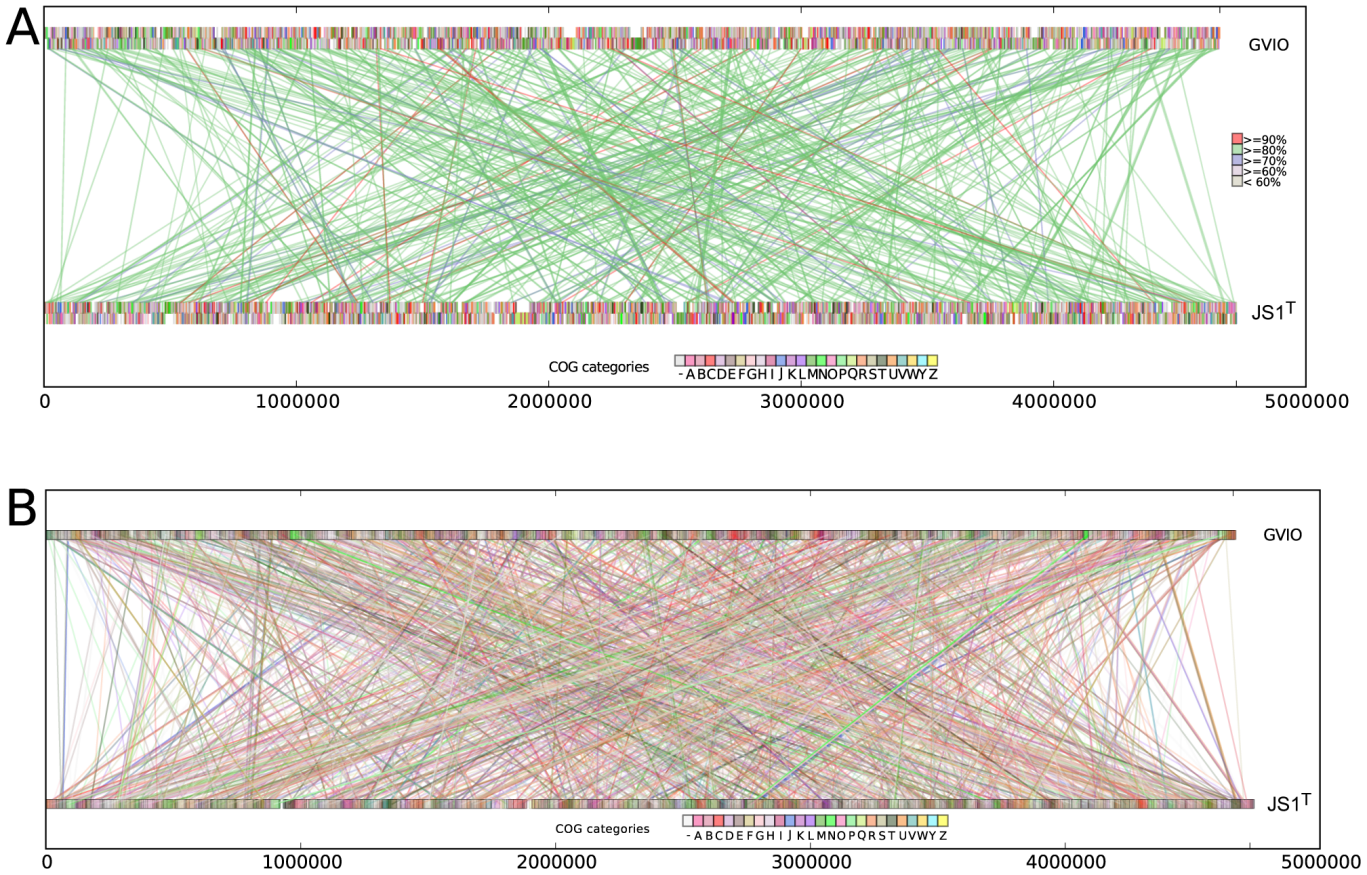
Comparison of gene synteny and genome rearrangements between *Gloeobacter kilaueensis* JS1^T^ and *Gloeobacter violaceus* PCC 7421^T^. (A) MUMMER alignment; colored rectangular blocks represent protein coding sequences according to COG functional categories. Lines represent matching DNA segments between the two genomes. Colors of connecting line segments are categorized according to % identities. (B) Shared orthologs identified between *Gloeobacter kilaueensis* JS1^T^ and *Gloeobacter violaceus* PCC 7421^T^ and their locations in the genomes. Lines connect orthologous genes and are colored according to COG functional categories. BLASTp E-values between bi-directional best hits are less than or equal to 1×10^−5^. Custom Python scripts were written to visualize these alignments.

Based on a 16S rRNA gene nucleotide identity of 1465/1485 nt (98.7%) between JS1^T^ and GVIO, these strains might generally be considered to belong to the same *G. violaceus*. Having only a non-axenic culture of JS1^T^, however, precluded *in vitro* DDH of JS1^T^ and GVIO. The complete genome sequences of JS1^T^ and GVIO were thus compared *in silico*, the result of which suggested JS1^T^ and GVIO do not belong to the same species. The JS1^T^ and GVIO genomes were compared in ANI to calculate percent identities between genomic DNA fragments [33]. Using the JSpecies program with default parameters [34], ANI between the JS1^T^ and GVIO genomes was 73.75% (with BLAST) and 83.11% (with MUMMER), well below the 90% cut-off value for species delineation by this approach. We then calculated *in silico* DDH values using the Genome-to-Genome Distance Calculator (GGDC) with three formulae [36]; GGDC calculations with BLAST revealed DDH values of 11.3%, 13.5%, and 8.72%. In MUMMER, DDH between JS1^T^ and GVIO was 14.96%. Both methods gave DDH values well below the cut-off of 60*%* for delineation of species. Also, little synteny existed between the JS1^T^ and GVIO genomes. Both genomes were aligned in MUMMER with default parameters, and the alignment plots were visualized with a custom Python script (Figs. 3A, B). Sequence identities between the two genomes averaged 83.4%. Matching segments were small (average 1 kbp; largest 6.1 kbp) and scattered throughout the genome, rather than in the large, conserved syntenic blocks one might see in closely related bacteria species. These data suggest JS1^T^ and GVIO differ markedly at the genome level.

### *Gloeobacter* phylogeny and evolution

**Figure 4:**
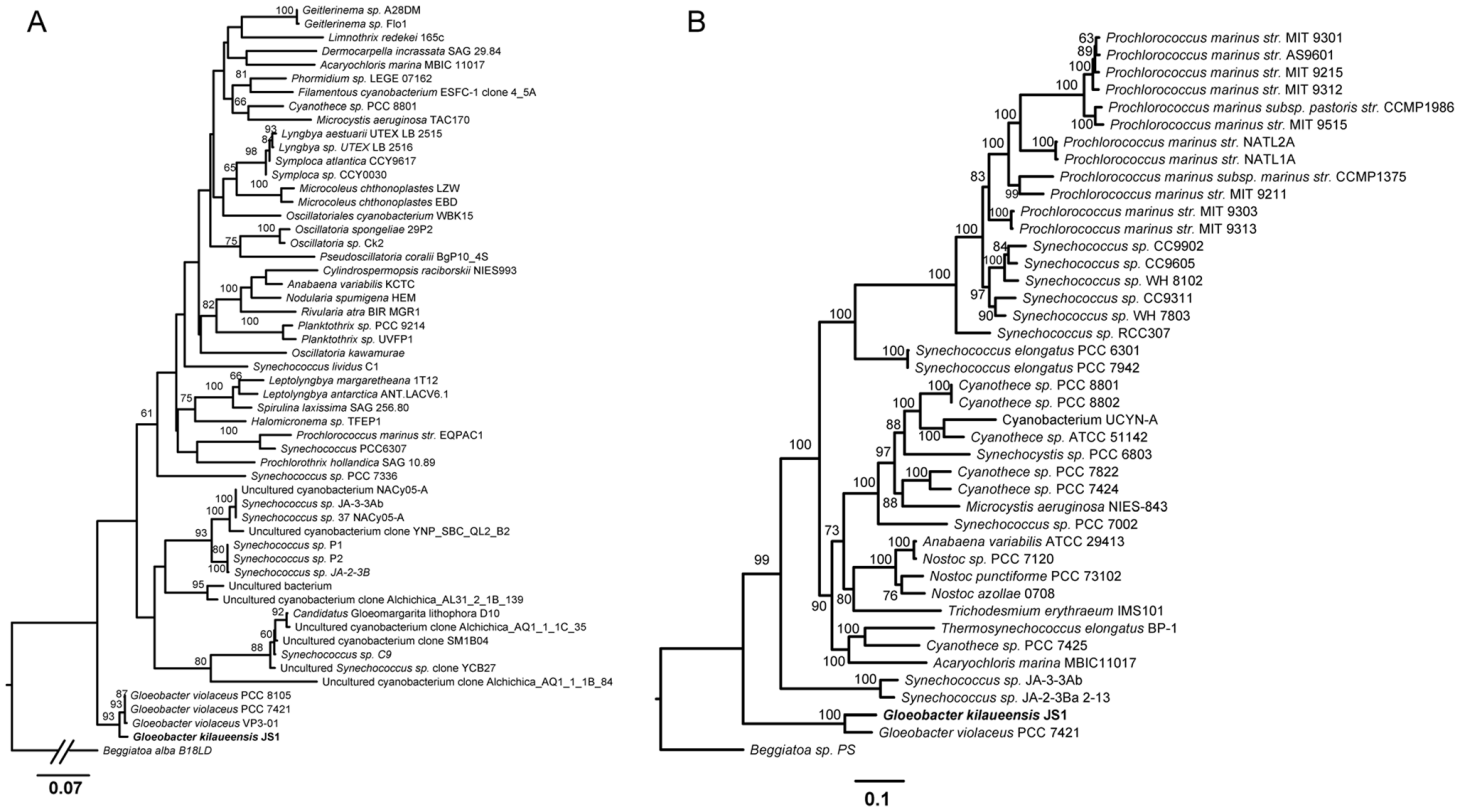
Maximum likelihood phylogenetic tree based on 16S rRNA gene sequences. Sequences were aligned using Muscle, edited with Gblocks, and the tree was inferred using RAxML program with 100 bootstrap replicates and the GTR+Gamma model of rate substitution. The root of the tree was shortened to fit the figure (A). Sequences in this tree included the top 50 BLASTn matches of the *Gloeobacter kilaueensis* JS1^T^ 16S rDNA sequence, among others. A phylogenetic tree was also constructed on the basis of 43 ribosomal proteins found in 41 cyanobacteria and the *Beggiatoa* sp. PS outgroup (B). Amino acid sequences of the 43 such proteins in each genome were individually aligned using Muscle, trimmed with Gblocks, and concatenated. Based on 5,359 aligned characters, the maximum likelihood analysis was performed using the RAxML program with the Gamma+WAG model of amino acid substitution and 100 bootstrap replicates, and visualized in FigTree.

JS1^T^ shares 98.7%, 98.6%, and 98.6% 16S rDNA sequence identity with *G. violaceus* strains PCC 7421^T^, VP3-01, and PCC 8105, respectively. A maximum likelihood phylogenetic tree based on cyanobacteria 16S rDNA sequences and with *Beggiatoa alba* B18LD as an outgroup, shows GVIO placing deeper along the cyanobacteria lineage than JS1^T^ (Fig. 4A). Outgroup selection affects phylogenetic tree topology, and *Beggiatoa* was used as the outgroup here because it has the shortest distance to the cyanobacteria clade and thus gives a more accurate tree topology than other outgroups, resulting in *Gloeobacter* near the root of the cyanobacterial lineage [37]. Though limited in nucleotide variability, the availability of 16S rDNA sequences from a vast number of cyanobacteria allows the evolutionary lineage of the *Gloeobacter* clade to be traced. Some sequences in Couradeau et al. [38] were included to determine if the intra-cellular carbonate forming cyanobacteria clade (*Candidatus* Gloeomargarita lithophora) branches more deeply than the *Gloeobacter*. The tree shows the clade including ‘*Gloeomargarita*’ forms a group distinct from *Gloeobacter* and closer to thermophilic *Synechococcus* spp. than to *Gloeobacter*. To better resolve the lineage of JS1^T^ in the cyanobacteria, amino acid sequences of 43 ribosomal proteins in 41 cyanobacteria (including JS1^T^) genomes and the *Beggiatoa* sp. PS outgroup were aligned, trimmed and concatenated, and a maximum likelihood phylogenetic analysis was performed using RAxML. The result of this analysis, visualized in FigTree, placed GVIO closer to the root than JS1^T^ (Fig. 4B). Previous whole-genome phylogenetic trees of cyanobacteria genomes largely agree with this tree's topology [37], [39], [40].

### Divergence time of *G. kilaueensis* JS1^T^ and *G. violaceus* from their last common ancestor

Divergence time between cyanobacteria species was estimated by Bayesian analysis in BEAST version 1.7.5 [41]. As phylogenomic approaches using multiple genes may be prone to errors introduced by genes, which are frequently, transferred (through horizontal gene transfer), only the 16S rRNA gene was used in this analysis. A recent study revealed that evolution of multicellularity in cyanobacteria coincided with increased diversification of the cyanobacteria clade during or at the beginning of the Great Oxidation Event [42]. The same study also estimated the divergence times of major clades in the *Cyanobacteria* phylum. To date the divergence time of JS1^T^ and GVIO, the JS1^T^ 16S rRNA gene sequence was aligned with those of taxa used by Schirrmeister *et al.* [42] and the analysis was repeated. Our BEAST analysis produced divergence time estimates very similar to those in the original study, specifically, an age of 2.52 Gya at the node (Node 3) leading to the first multicellular cyanobacteria, and an age of 1.77 Gya at the node (Node 31) leading to first terminally differentiated cyanobacteria (Fig. S4). Node age where JS1^T^ and GVIO diverged was estimated at ∼283 million years, *i.e.*, during the Mesozoic era (Fig. S4).

To map genes gained and lost along the cyanobacteria lineage, phyletic patterns were compiled based on the presence or absence of 13,655 orthologous genes identified in the 41 cyanobacteria genomes mentioned above. These phyletic patterns were uploaded to the Gain Loss Mapping Engine (GLOOME) server [43] to calculate gene gain and loss events using a stochastic mapping approach [44]. The goal is to detect genes gained or lost during the evolutionary history of the last common ancestor of the two *Gloeobacter* from which two *Gloeobacter* species emerged. This revealed that JS1^T^ gained 493 and lost 363 genes from the node branching off from GVIO (Fig. S5).

### Comparative genomic analyses

Little synteny exists between the JS1^T^ and GVIO genomes, despite their sharing >98% 16S rDNA gene sequence identity (cf. Figs. 3A, B). In closely related bacterial species or strains, gene synteny is usually conserved, and would be evidenced by large blocks of colinear genomic regions. The genome of JS1^T^ appears to have undergone considerable rearrangement.

**Figure 5:**
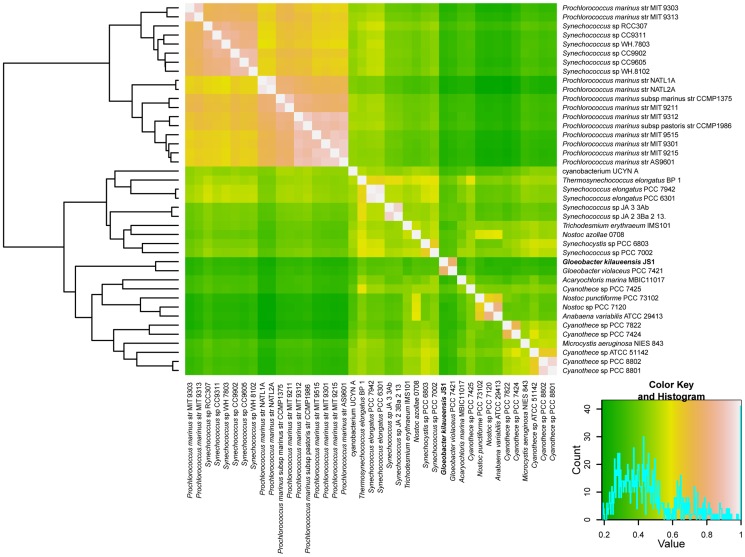
Hierarchical clustered heatmap representation of completely sequenced cyanobacteria genomes based on a correlation matrix of the presence or absence of 13,655 orthologous groups identified in 41 cyanobacteria. The 13,655×41 matrix was imported into the R program, and Pearson correlation coefficients were calculated using the ‘gplots’ package. Colors represent correlation values ranging from 0 to 1, with green representing lowest correlation and white representing highest correlation.

Using orthologous groups identified by the OrthoMCL program, the presence or absence of these orthologous groups was counted in each of the 42 cyanobacteria genomes mentioned above. This was recorded in a 13,655×42 matrix of ‘1’s and ‘0’s, where ‘1’ represents presence, and ‘0’ represents absence. A Pearson correlation coefficient was calculated for this matrix in the R statistical analysis tool, and viewed as a clustered heatmap (Fig. 5). This approach has been useful in understanding niche specialization by comparing complete genomes of *Bacteroidetes* adapted to different lifestyles [45]. Distinct clusters of different strains of marine *Prochlorococcus* and *Synechococcus*, and freshwater *Synechococcus* can also be visualized (*e.g.*, Fig. 4). Similarly, JS1^T^ and GVIO grouped tightly in a cluster separate from other cyanobacteria. Despite the two genomes having gone through large-scale rearrangements, however, they still share a large number of orthologous groups, and seem able to perform similar functions based on comparison of orthologous groups of genes.

### Recruitment of *Gloeobacter* reads from the cave epilithic biofilm metagenome

The assembled JS1^T^ genome was used to recruit *Gloeobacter*-specific reads from the previously generated epilithic biofilm metagenome (Brown *et al.*, unpubl.). Recruitment using the NUCMER script from the MUMMER aligner identified 3,474 unique metagenomic reads (20,433 unique reads with BLASTn using relaxed parameters). Conversely, only 596 unique reads (19,101 reads with BLASTn using relaxed parameters) were recruited when the *Gloeobacter violaceus* PCC 7421^T^, genome was used as the reference. BLASTn parameters were relaxed to recruit sequences with identities as low as ∼60*%* so reads from distantly related organisms might be recovered. Recruitment plots using GVIO yielded mostly reads that have <90*%* nucleotide identity (Figs. S6A, S6B). These findings underscore the need for more reference genomes in public databases, because even at 98.7*%* 16S rDNA nucleotide identity, GVIO is not the most ideal organism to extract from the sample all metagenomic sequences that affiliate with *Gloeobacter*. This also emphasizes the need for more reference genomes of rare but nevertheless important organisms.

## Conclusions

*G. kilaueensis* sp. nov., the first new *Gloeobacter* species to be described in almost forty years, was cultivated from a lava cave in volcanically active Kīlauea Caldera, Hawai'i. The type strain of *G. kilaueensis* sp. nov. is JS1^T^. The complete genome of JS1^T^ was sequenced based on DNA extracted from an enriched non-axenic culture resembling a low-complexity metagenome. *In silico* DDH analyses revealed JS1^T^ is distinct from the only other known species in the genus, *Gloeobacter violaceus* PCC 7421^T^. Despite the genomes of JS1^T^ and PCC 7421^T^ (GVIO) sharing little synteny, the number of genes in each is comparable, and they share more orthologous genes with each other than either does with other fully sequenced cyanobacteria. Phylogenetic trees (16S rRNA and 43 concatenated ribosomal proteins) placed JS1^T^ in the same deep-branching, monophyletic clade as PCC 7421^T^, but PCC 7421^T^ appears more deeply branching. A divergence time estimate using 16S rRNA gene sequences determined that JS1^T^ and PCC 7421^T^ (GVIO) separated approximately 283 million years ago.

### Description of *Gloeobacter kilaueensis* sp. nov

*Gloeobacter kilaueensis* (ki.laue.en'sis N.L. masc. adj. kilaueensis), of or pertaining to Kīlauea, the area from which the type strain was isolated. Colonies on modified BG-11 medium are dark purple, smooth, shiny, raised to high convex, and opaque. Cells stain Gram negative, and are rods of ∼3.5×1.5 µm. Purple colonies do not develop on BG-11 incubated in darkness at 30°C, but the strain appears obligately photoautotrophic, with growth under weak illuminance (∼500 lx). Thylakoid membranes are absent. Growth occurs on modified BG-11 at 27 and weakly at 37°C. The cells are neither motile, nor do they glide. Major pigments are chlorophyll *a* and ß-carotene. The type strain of *G. kilaueensis* is JS1^T^ ( = ATCC BAA-2537 = CCAP 1431/1 = ULC0316. The holotype of *G. kilaueensis* is in the Algal Collection of the US National Herbarium (US# 217948). JS1^T^ was isolated from an epilithic biofilm on the wall of a lava cave in volcanically Kīlauea Caldera on the island of Hawai'i. The type strain's DNA G+C mol content is 60.54%.

## Materials and Methods

### Ethics Statement

Fieldwork and sampling was conducted in Kīlauea Caldera, Hawai'i, under permit no. HAVO-2009-SCI-0029 issued to SPD by the Hawai'i Volcanoes National Park (National Parks Service, Department of the Interior).

### Sampling and Cultivation

Part of a purple-pigmented epilithic biofilm was aseptically transferred from a lava cave wall in Kīlauea Caldera, to a sterile 1.5 mL cryogenic tube. The sample was taken to a laboratory within 12 hours, whereupon it was aseptically dissected with a sterile scalpel. Sub-samples were aseptically transferred to a modified BG-11 medium in a culture tube, and shaken at 200 rpm in a 2% CO_2_ atmosphere in a light-incubator. The modified BG-11 medium contained 1.5 gL^−1^ NaNO_3_, 0.036 gL^−1^ CaCl2•2H_2_O, 0.012 gL^−1^ FeNH_4_ Citrate, 0.001 gL^−1^ Na_2_EDTA, 0.02 gL^−1^ K_2_HPO_4_, 0.075 gL^−1^ MgSO4•7H_2_O, 0.02 gL^−1^ Na_2_CO_3_, and micronutrients (Haagland's). The culture tube was wrapped in a white paper tissue to reduce illuminance to match that in the cave entrance (∼480 lx; ∼45 fc; ∼6.5 µE m^−2^ sec^−1^). Purple flocs developed after about two weeks; 10 µl of this cell suspension was spread on solid BG-11. Raised convex, dark purple, mucoid colonies developed on BG-11 after one week. A Pasteur pipette drawn to a fine point was used to transfer ‘spots’ of the purple colonies to a fresh BG-11 plate to isolate single colonies. Dark purple colonies developed on this medium after two weeks of incubation.

### Genomic DNA Extraction and Quality Control

Since an axenic culture of JS1^T^ was not available, genomic DNA from an enriched culture was extracted and sequenced. Genomic DNA was extracted from approximately 1 g wet wt. of JS1^T^ cells, including a small percentage of cells of at least two heterotrophic bacteria, in the MoBio Ultraclean® Soil DNA isolation kit. This gDNA was used to construct a 16S rRNA gene clone library through which the identity of DNA from other organisms was assessed. The Zero Blunt® PCR Cloning Kit (Life Technologies, Carlsbad, CA) was used to construct the 16S rRNA gene clone library with primers 27F and 1492R [46] in 50 µL PCRs containing 5 µL of 10× *Pfu* Buffer, 1 µL of 10 µM dNTP mixture, 5 µL of *Pfu* DNA polymerase, 1 µL of 10 mM primer, 1 µL of DNA template, and nuclease-free water. PCR conditions were 95°C (5 min), followed by 35 cycles of 95°C (30 sec), 52°C (30 sec), 72°C (30 sec), and a final extension of 72°C (7 min). Amplifications were performed in a Bio-Rad Thermal Cycler (Bio-Rad Laboratories, Hercules, CA). PCR products were cleaned in the MoBio UltraClean® PCR Clean-Up Kit. Purified PCR products were cloned into pCR®-Blunt II-TOPO vector (Life Technologies, Carlsbad, CA) and transformed into chemically competent One Shot® TOP10 *E. coli* cells. Transformed cells were plated on LB+Kanamycin agar plates, then isolated and grown in Circle Grow® (Q-BIOgene, Carlsbad, CA). 42 cloned inserts were amplified and sequenced using M13F and M13R primers.

### Sequencing, Genome Assembly, and Finishing

gDNA extracted from the JS1^T^ culture as described above was prepared for sequencing in the Advanced Studies in Genomics, Proteomics, and Bioinformatics Center (ASGPB) at the University of Hawai'i. An 8 kb paired-end 454 library was prepared according to the Roche protocol and sequenced in a 454 GS-FLX Titanium sequencer (454 Life Sciences, Branford, CT). This provided 222,335 sequences, comprising 155,068 paired-end sequences and 66,513 singletons, *i.e.*, a total of 221,581 usable sequences. The remainder was discarded due to their poor quality. Paired-end Illumina sequences were also generated in an Illumina Genome Analyzer *IIx* (Illumina Inc, San Diego, CA), and comprised 4,792,504 sequences (2,396,252 paired-end sequences). After trimming for quality, 4,756,989 of the original sequences were used in assembly or read recruitment.

Raw sequences produced by Roche 454 GS FLX sequencer were first assembled using Newbler version 2.6. The MUMMER sequence alignment tool [11] used assembled Newbler contig scaffolds to recruit sequences produced by the Illumina Genome Analyzer *IIx*. Each Newbler contig scaffold was then assembled with quality-trimmed Illumina reads using the Minimus assembler in the AMOS package. Often, coverage of Illumina reads was found to be more than required for quality improvement, so a Python script was written to only recruit about 15 additional reads where needed to improve the quality in otherwise low quality regions. This procedure improved and corrected the quality of the Newbler assembled contigs, those that initially contained only 454 pyrosequences. Illumina sequences also helped to correct ambiguous sequence regions caused by homopolymers present in 454 reads.

Pyrosequences and Illumina sequences were assembled together by the Celera Assembler in order to compare those contigs with the Newbler-assembled contigs. Celera contigs were then shredded into 500 bp fragments with 200 bp overlapping regions, and used in Minimus assemblies to close gaps. The final gaps between quality-improved and Minimus-assembled contigs were manually closed using Seqman (DNAstar Inc, Madison, WI). Illumina reads were used first to close gaps, and where gaps remained, primers were designed to amplify the gap regions for sequencing in an ABI3730xl (Life Technologies, Carlsbad, CA). The error rate of the final assembled genome is less than 1 in 100,000. Illumina and 454 sequences provided roughly 93× coverage of the genome, *i.e.*, 440,800,613 bases. A circular representation of the genome was drawn in Circos [47].

### Verification of genome assembly

Trimmed 454 pyrosequences were taxonomically assigned using the PhymmBL binning tool [13]. Mate pairs with at least one read belonging to the genus *Gloeobacter* were aligned against the assembled genome using the MUMMER alignment tool, and overlapping paired-ended reads binned as *Gloeobacter* were graphically represented in tiling paths with a custom Python script. The algorithm selecting mate pairs searched those spanning a given segment of the genome for ‘*Gloeobacter*’-binned reads, and where they were found, only pairs fitting the expected insert size (5,000–12,000 bp) were reported.

Contig scaffolds produced by Newbler and Celera assemblers were also aligned against the assembled genome to determine if mis-assembly may have occurred. PCR amplification of suspicious boundaries were performed where G+C content varied significantly from the rest of the genome, *i.e.*, less than the mean of 60.5*%* and where the coverage of reads binned as *Gloeobacter* fell and reads from other organisms dominated.

Primers were designed by a custom Python script utilizing the Primer3 program [48] to have primers meeting the criteria needed for long-range PCR. Primers were designed to amplify approximately 15 kb fragments. The Qiagen® LongRange PCR kit was used to amplify the suspicious genome segments from the genomic DNA isolated from a non-axenic but largely *Gloeobacter* culture.

### Genome Annotation

Putative coding regions in the genome were identified in the Prodigal gene finder program [49] and submitted to the NCBI submission check tool to curate ORF start sites, and to identify frameshifts and gene fragments. ORFs with partially conserved domains were individually inspected to determine if the products are functionally inactive, and were assigned as pseudogenes where necessary. ORFs were searched against the NCBI Refseq database using BLASTp [50], and top hits were checked against the Protein Clusters database from NCBI to assign names to ORFs. Intergenic regions were extracted and searched against the Refseq database using BLASTx to identify potential coding regions missed by gene finders, and manually assigned.

### Phylogenetic Analyses

The 16S rRNA gene sequences of JS1^T^ and taxa determined by BLASTn searches to be closely related to JS1^T^ were aligned in Muscle [51], edited with Gblocks, and a maximum likelihood tree was built using the RAxML program [52]. The tree also contains several clones from a phylogenetic tree built by Couradeau et al. [38] to visualize placement of the *Gloeobacter* clade. For the ribosomal protein tree, all such proteins identified in JS1^T^ were searched through a bi-directional BLASTp search against the 40 cyanobacteria and *Beggiatoa* sp. PS genomes. Forty-three common ribosomal proteins were found in these genomes; protein sequences were aligned using Muscle, trimmed with Gblocks, and concatenated. A maximum likelihood analysis was then performed using RAxML, and the resulting phylogenetic tree was visualized in FigTree.

Divergence time estimates for selected cyanobacteria were calculated by Bayesian inference of an alignment of the 16S rRNA gene sequences from 58 cyanobacteria used in the analysis by Schirrmeister *et al.* [42], plus that of JS1^T^. Based on analysis 1 by Schirrmeister *et al.* [*ibid.*], 16S rRNA genes of the 59 cyanobacteria taxa were first aligned using mafft-linsi tool version 7.012b [53]. Gaps in the alignment were removed with the Trimal tool [54]. The final alignment contains 1090 characters without gap characters. Bayesian analysis was carried out with BEAST version 1.7.5 [41] with the following parameters: General Time Reversible model of nucleotide substitution with gamma distribution (GTR+G), relaxed clocks with uncorrelated lognormal distribution, and three calibration points using calibration points as described by Shirrmeister *et al.* [42]. Markov Chain Monte Carlo (MCMC) analysis using BEAST was conducted for 50 million generations, sampling every 2000^th^ generation. A consensus tree was obtained by the TreeAnnotator tool in the BEAST software package, with 25% burn-in of trees obtained from the BEAST analysis.

### Metagenome Recruitment

Metagenome reads were recruited in MUMMER with the following parameter: -minmatch 10 and using BLASTn with the following parameters:
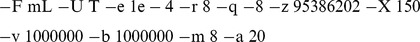



### Genome-to-genome distance and average nucleotide identities to resolve the *Gloeobacter* lineage

To determine if JS1^T^ might be considered a new *Gloeobacter* species, an *in silico* DNA-DNA Hybridization (DDH) was conducted with a Genome-To-Genome sequence comparison [36] and Average Nucleotide Identities (ANI) [33]. To calculate genome-to-genome distances, the complete genome sequence was uploaded with that of the reference *Gloeobacter violaceus* to the GGDC website (http://ggdc.gbdp.org/). Average nucleotide identities (ANI) between JS1^T^ and GVIO were calculated in the Jspecies program [34].

### Comparative genomic analyses

Completely sequenced genomes of 40 cyanobacteria (as of March 3, 2012) for use in comparative genomic analyses were downloaded from the NCBI website. Several of the cyanobacteria have multiple amplicons, so these were pooled into a single data set for each genome. Local BLAST databases of amino acid sequences for each genome were created by the ‘formatdb’ command (BLAST package) and all-vs-all BLASTp searches were conducted to create all possible combinations of relationships between all the amino acid sequences. Next, a MySQL database to host these results was created. BLAST results were loaded to the custom database, and orthologous groups between 41 cyanobacteria genomes were identified using scripts provided by the OrthoMCL program [55]. Whole genome alignment between JS1^T^ and GVIO was performed in the MUMMER program and visualized through a custom Python script. Visualization of orthologous genes between JS1^T^ and GVIO along the genome coordinates was also completed through another custom Python script.

### Analysis of chlorophyll and carotenoid pigments by HPLC

Purple colonies collected from the surface of BG-11 plates were extracted in HPLC-grade acetone at 4°C for 24 hours. The extracts were then brought to room temperature, vortexed, and centrifuged for 5 minutes to remove cellular debris. Mixtures of 1 mL extract plus 0.3 mL HPLC grade water were prepared in opaque autosampler vials, and 200 µL injected onto a Varian 9012 HPLC system equipped with a Varian 9300 autosampler, a Timberline column heater (26°C), and a Waters Spherisorb® 5 µm ODS-2 analytical (4.6×250 mm) column and corresponding guard cartridge (7.5×4.6 mm). Pigments were detected with a ThermoSeparation Products UV2000 detector (λ_1_ = 436, λ_2_ = 450). A ternary solvent system was used for pigment analysis: Eluent A (methanol:0.5 M ammonium acetate, 80∶20, v/v), Eluent B (acetonitrile∶water, 87.5∶12.5, v/v), and Eluent C (100*%* ethyl acetate). Solvents A and B contained an additional 0.01*%* 2,6-di-ter-butyl-*p*-cresol (0.01% BHT, w/v; Sigma-Aldrich) to prevent the conversion of chlorophyll *a* into chlorophyll *a* allomers. The linear gradient used for pigment separation was modified from Wright et al. (1991): 0.0' (90% A, 10% B), 1.00' (100% B), 11.00' (78% B, 22% C), 27.50' (10% B, 90% C), 29.00' (100% B), 30.00' (100% B), 31.00' (95% A, 5% B), 37.00' (95% A, 5% B), and 38.00' (90% A, 10% B) [56]. Eluent flow rate was held constant at 1.0 mL min^−1^. Pigment peaks were identified by comparison of retention times with those of pure standards and extracts prepared from algal cultures of known pigment composition.

## Supporting Information

Figure S1 Autofluorescent *Gloeobacter kilaueensis* JS1^T^ cells observed under bright field (A) and fluorescent microscopy (B). Both fields recorded through a 63× objective with oil immersion on a Zeiss PALM Laser Capture Microdissection MicroBeam IV system. Dividing cells are visible. Scale bar = 10 µm.
(TIF)

Figure S2 Genome assembly verification plot for *Gloeobacter kilaueensis* JS1^T^. Contigs produced by Celera and Newbler assemblers are aligned against the finished genome and shown as a gold line near the bottom of the top panel. Consistent (∼9 kb) mate pairs identified as *Gloeobacter* in origin and aligned against the finished genome are plotted as black line segments, and appear here as continuous black lines across the genome because of the close proximity of mate pairs). Singleton reads binned as *Gloeobacter* are shown as blue line segments. Suspicious regions with low G+C% are highlighted as beige rectangles. Bottom panel: G+C% for a given 1,000 bp region along the genome, as blue lines. Also shows coverage of reads binned as either *Gloeobacter* in origin or not. Reads binned as *Gloeobacter* are purple, while others are green.
(TIF)

Figure S3 Agarose gel showing long range PCR products (∼15 kb). The two left and right outermost lanes are DNA markers (left: λ marker, right: 1 kb marker). Genomic regions whose G+C mol% is <60% were amplified using primers designed for long-range PCR to confirm presence of such regions the JS1^T^ genome.
(TIF)

Figure S4 MCMC tree showing divergence times in the cyanobacteria lineage. The tree was built using 16S rRNA genes from 59 cyanobacterial taxa including JS1^T^ as in the study by Schirrmeister *et al.* [42]. Numbers near the nodes specify approximate divergence time in billion years. Node where JS1^T^/GVIO split occurred is indicated with a red asterisk and the two nodes (Node 3 and Node 31) from Schirrmeister *et al.* are highlighted in red. The two *Gloeobacter* species are highlighted in purple.
(TIF)

Figure S5 Gene gain/loss events in the cyanobacteria lineage. Phylogenetic tree built by stochastic mapping of phyletic patterns representing gene gains or losses. Scale bar represents the number of gain events, and branch length represents gain events. Numbers in blue indicate gene gains, those in red indicate gene losses.
(TIF)

Figure S6 Recruitment plots of metagenomic reads using (A) *Gloeobacter kilaueensis* JS1^T^ and (B) *Gloeobacter violaceus* PCC 7421^T^ genomes as references. Each colored dot represents a positive BLASTn match (red ≥90%, green ≥80%, blue ≥70%, lavender ≥60%, grey <60%). Dark green histograms indicate average number of reads recruited per 1,000 bp sliding window.
(TIF)

Table S1 Genes in the five ‘suspicious’ regions identified in the JS1^T^ genome. See text for further details.
(XLSX)

Table S2 CRISPR repeats in the JS1^T^ genome, and the number of spacers in each.
(XLSX)
