# Supplementary figures and images for: Cultivation and Complete Genome Sequencing of Gloeobacter kilaueensis sp. nov., from a Lava Cave in Kīlauea Caldera, Hawai'i
Source: PLoS One. 2013 Oct 23;8(10):e76376. doi: 10.1371/journal.pone.0076376 (PMC3806779; doi:10.1371/journal.pone.0076376)

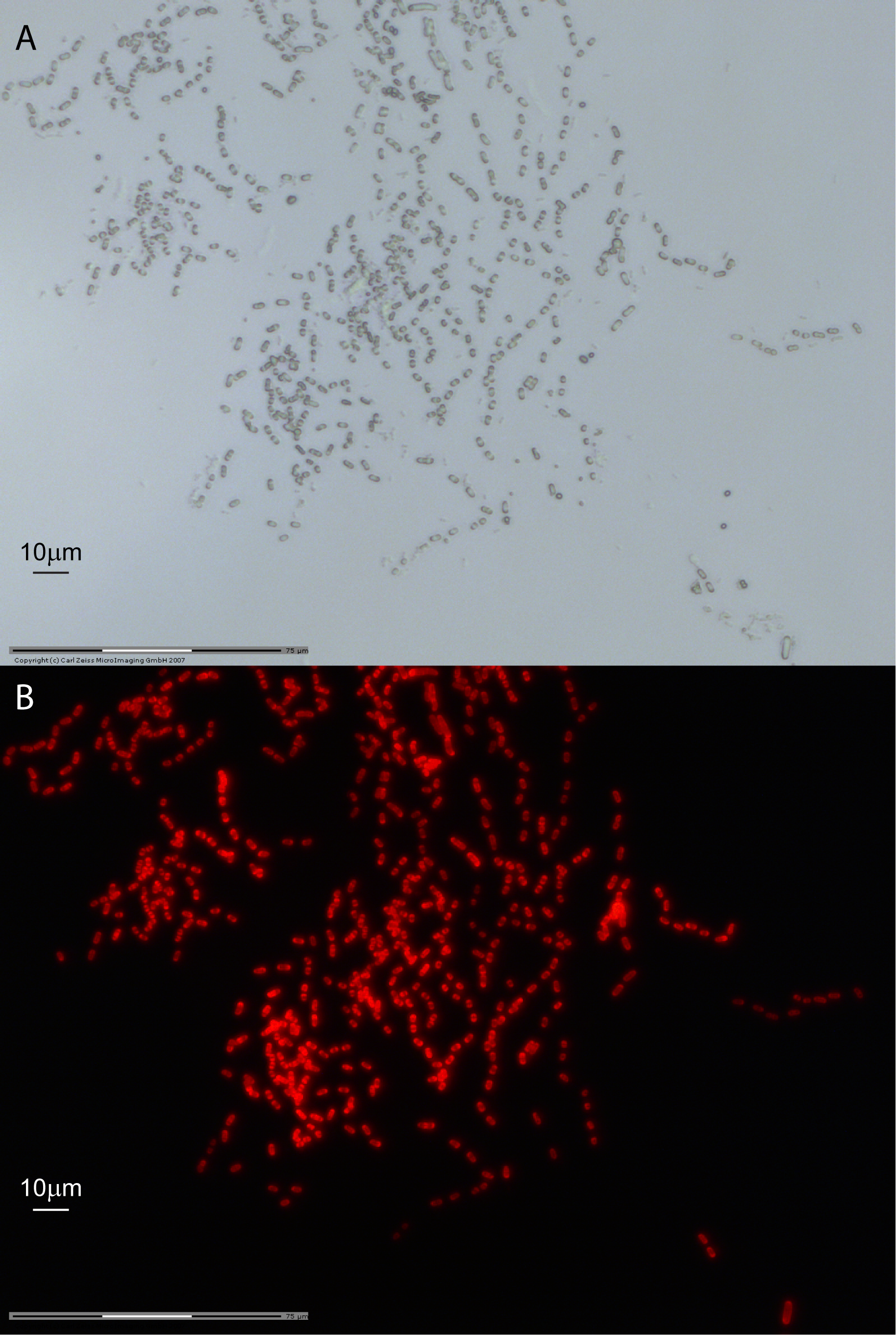

Supplement: Figure S1 — Autofluorescent Gloeobacter kilaueensis JS1T cells observed under bright field (A) and fluorescent microscopy (B). Both fields recorded through a 63× objective with oil immersion on a Zeiss PALM Laser Capture Microdissection MicroBeam IV system. Dividing cells are visible. Scale bar = 10 µm. (TIF) [file pone.0076376.s001.tif]

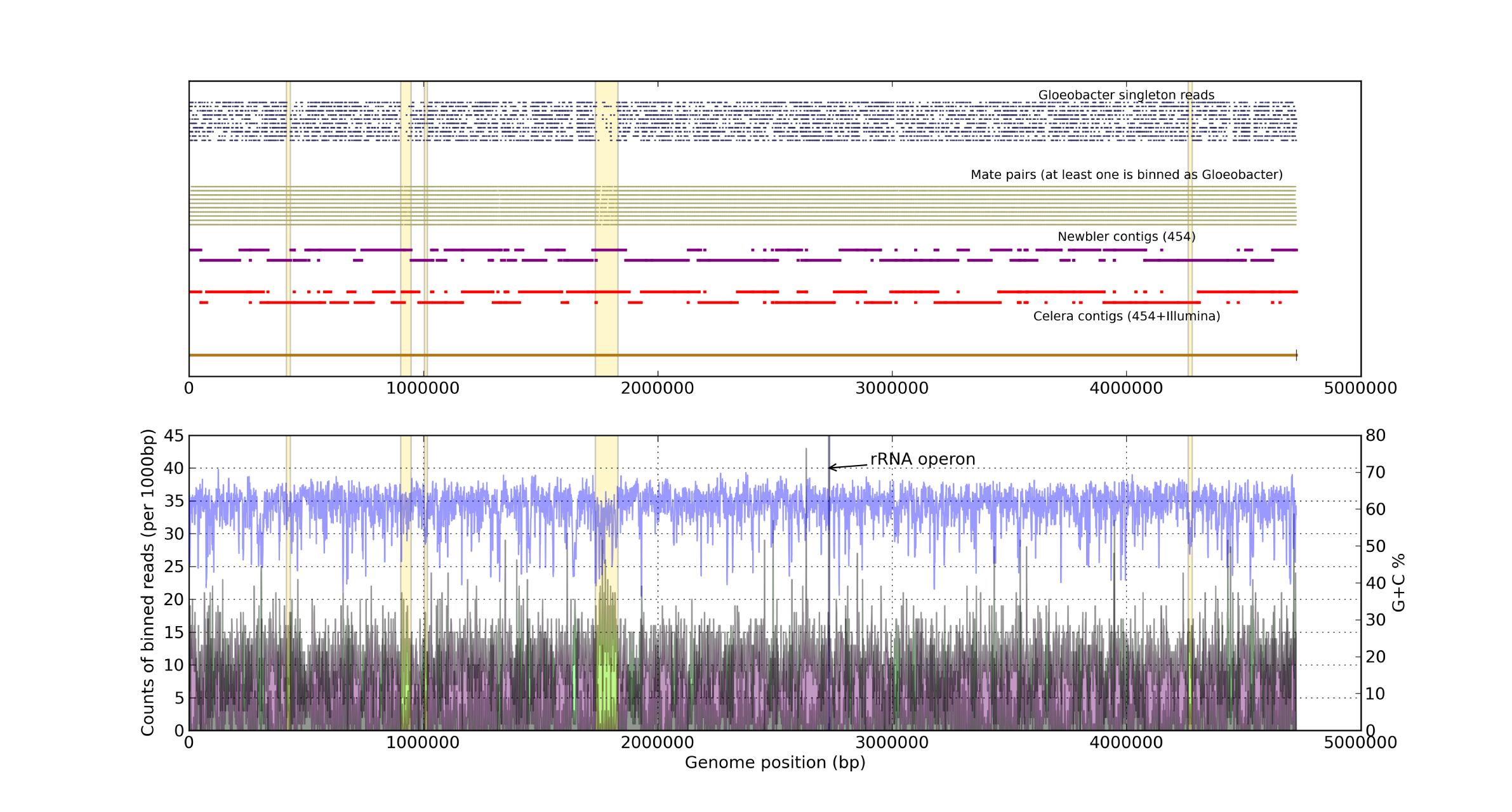

Supplement: Figure S2 — Genome assembly verification plot for Gloeobacter kilaueensis JS1T. Contigs produced by Celera and Newbler assemblers are aligned against the finished genome and shown as a gold line near the bottom of the top panel. Consistent (∼9 kb) mate pairs identified as Gloeobacter in origin and aligned against the finished genome are plotted as black line segments, and appear here as continuous black lines across the genome because of the close proximity of mate pairs). Singleton reads binned as Gloeobacter are shown as blue line segments. Suspicious regions with low G+C% are highlighted as beige rectangles. Bottom panel: G+C% for a given 1,000 bp region along the genome, as blue lines. Also shows coverage of reads binned as either Gloeobacter in origin or not. Reads binned as Gloeobacter are purple, while others are green. (TIF) [file pone.0076376.s002.tif]

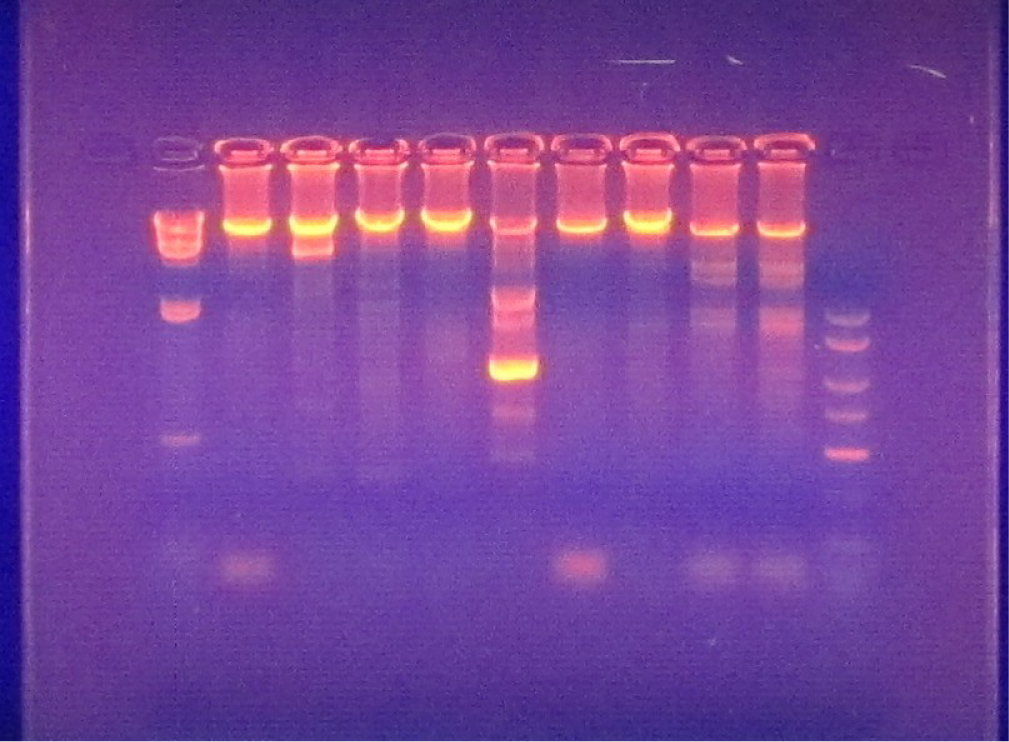

Supplement: Figure S3 — Agarose gel showing long range PCR products (∼15 kb). The two left and right outermost lanes are DNA markers (left: λ marker, right: 1 kb marker). Genomic regions whose G+C mol% is <60% were amplified using primers designed for long-range PCR to confirm presence of such regions the JS1T genome. (TIF) [file pone.0076376.s003.tif]

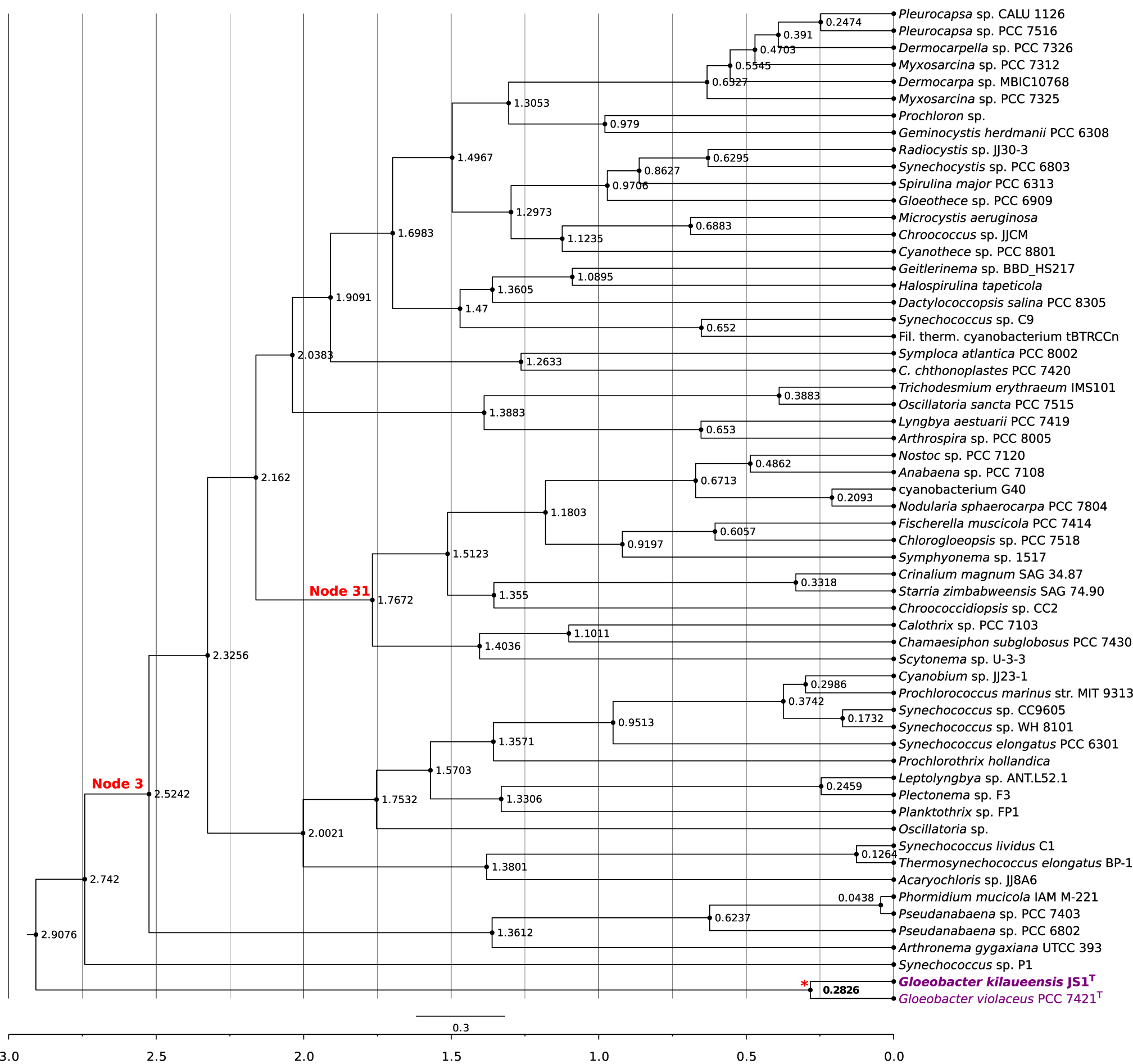

Supplement: Figure S4 — MCMC tree showing divergence times in the cyanobacteria lineage. The tree was built using 16S rRNA genes from 59 cyanobacterial taxa including JS1T as in the study by Schirrmeister et al. [42]. Numbers near the nodes specify approximate divergence time in billion years. Node where JS1T/GVIO split occurred is indicated with a red asterisk and the two nodes (Node 3 and Node 31) from Schirrmeister et al. are highlighted in red. The two Gloeobacter species are highlighted in purple. (TIF) [file pone.0076376.s004.tif]

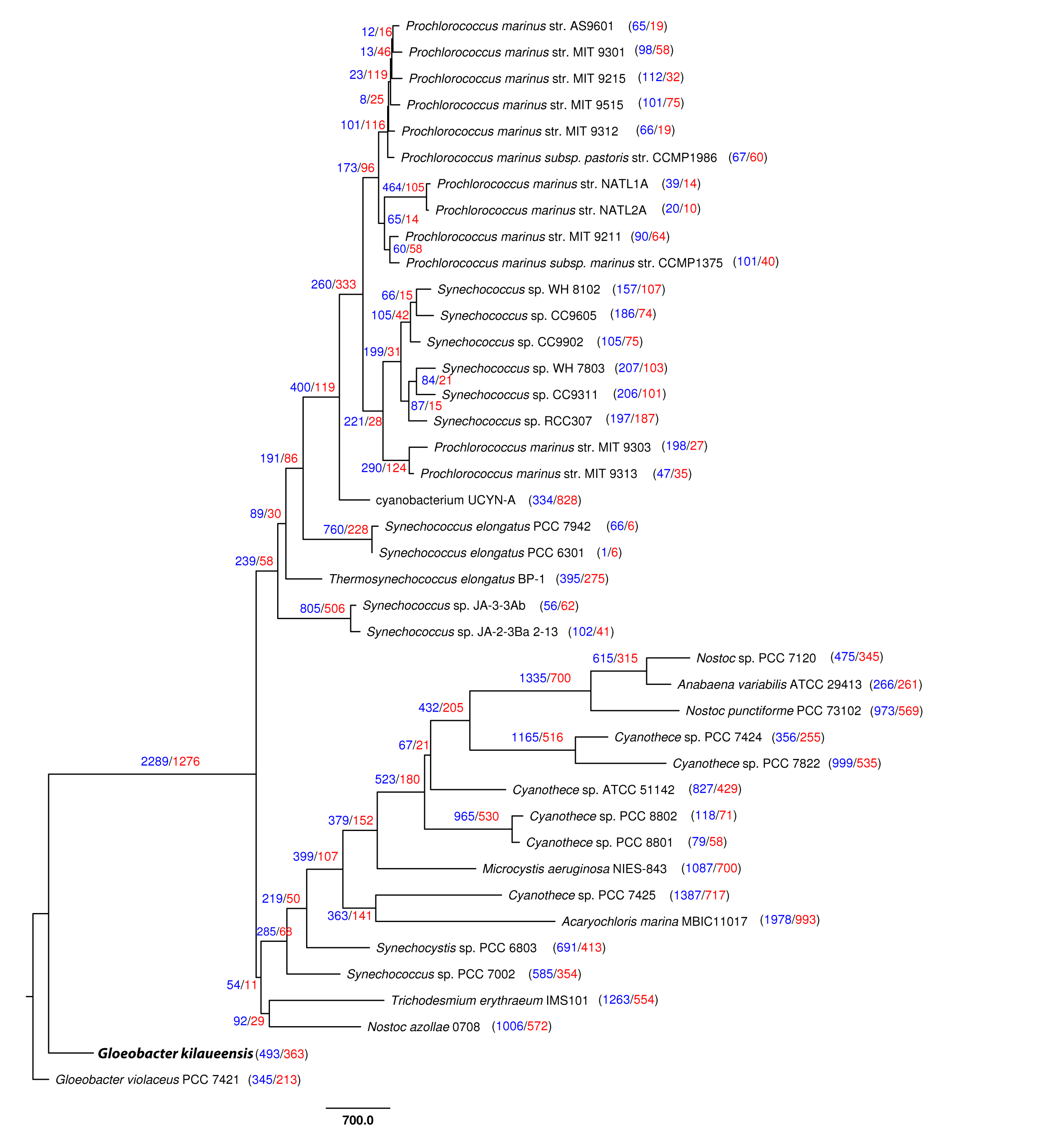

Supplement: Figure S5 — Gene gain/loss events in the cyanobacteria lineage. Phylogenetic tree built by stochastic mapping of phyletic patterns representing gene gains or losses. Scale bar represents the number of gain events, and branch length represents gain events. Numbers in blue indicate gene gains, those in red indicate gene losses. (TIF) [file pone.0076376.s005.tif]

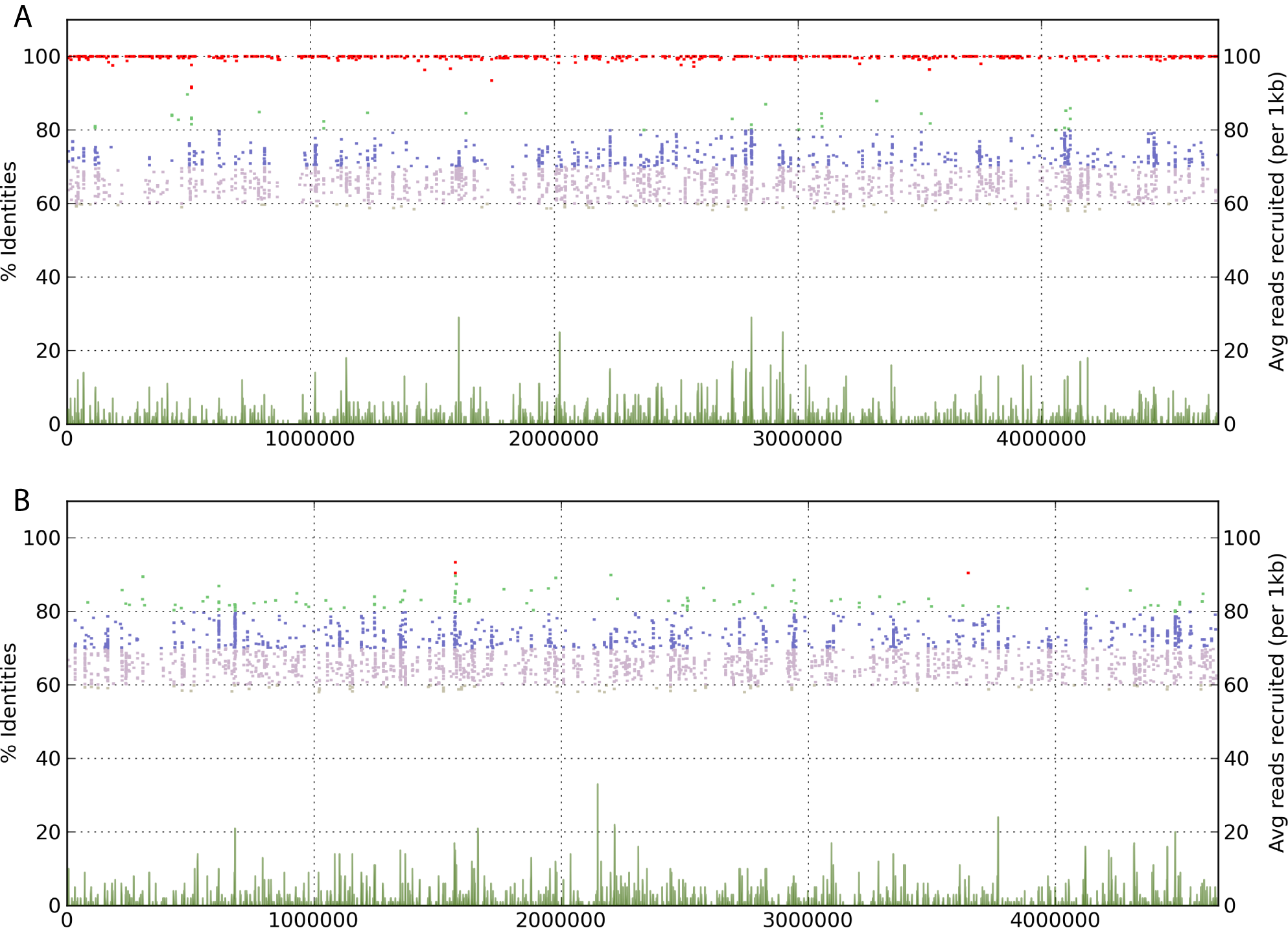

Supplement: Figure S6 — Recruitment plots of metagenomic reads using (A) Gloeobacter kilaueensis JS1T and (B) Gloeobacter violaceus PCC 7421T genomes as references. Each colored dot represents a positive BLASTn match (red ≥90%, green ≥80%, blue ≥70%, lavender ≥60%, grey <60%). Dark green histograms indicate average number of reads recruited per 1,000 bp sliding window. (TIF) [file pone.0076376.s006.tif]
